# Design of Aging Smart Home Products Based on Radial Basis Function Speech Emotion Recognition

**DOI:** 10.3389/fpsyg.2022.882709

**Published:** 2022-05-04

**Authors:** Xu Wu, Qian Zhang

**Affiliations:** ^1^School of Art and Design, Tianjin University of Technology, Tianjin, China; ^2^School of Control and Mechanical Engineering, Tianjin Chengjian University, Tianjin, China

**Keywords:** artificial intelligence, speech emotion recognition, HMM, RBF, dynamic optimal learning rate, aging users

## Abstract

The rapid development of computer technology and artificial intelligence is affecting people’s daily lives, where language is the most common way of communication in people’s daily life. To apply the emotion information contained in voice signals to artificial intelligence products after analysis, this article proposes a design based on voice emotion recognition for aging intelligent home products with RBF. The authors first aimed at a smart home design, and based on the problem of weak adaptability and learning ability of the aging population, a speech emotion recognition method based on a hybrid model of Hidden Markov/Radial Basis Function Neural Network (HMM/RBF) is proposed. This method combines the strong dynamic timing modeling capabilities of the HMM model and the strong classification decision-making ability of the RBF model, and by combining the two models, the speech emotion recognition rate is greatly improved. Furthermore, by introducing the concept of the dynamic optimal learning rate, the convergence speed of the network is reduced to 40.25s and the operation efficiency is optimized. Matlab’s simulation tests show that the recognition speed of the HMM/RBF hybrid model is 9.82–12.28% higher than that of the HMM model and the RBF model alone, confirming the accuracy and superiority of the algorithm and model.

## Introduction

**FIGURE 1:**
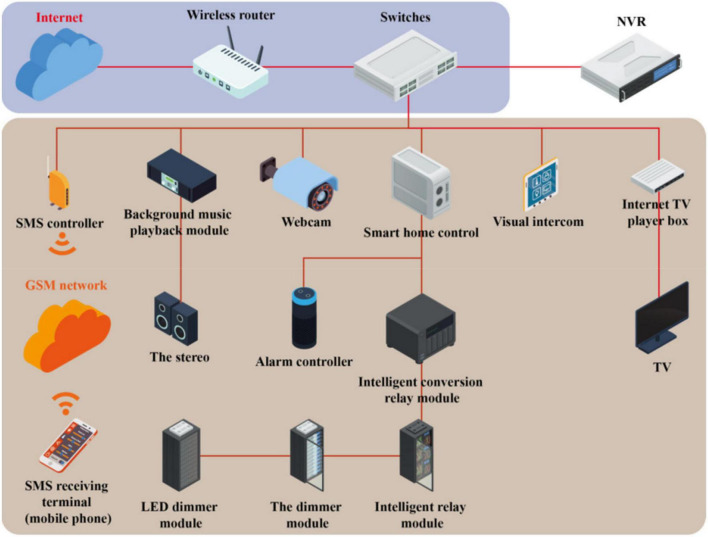
Schematic diagram of the artificial intelligence interaction process.

The rapid rise of artificial intelligence brings great convenience to people’s daily life. To make smart products more intelligent and humanized and the user experience more comfortable, the new human–computer interaction technology has gradually attracted the attention of the majority of researchers (Tao et al., 2021). Also, research by Reevess and Nass at Stanford University found that the key problem that “emotional intelligence” needs to solve is the ability of emotional intelligence; this is actually consistent with solving the problems of human–computer interaction and human-to-human communication (Peng et al., 2021). Therefore, to enable the computer actively and accurately perceive the user’s emotional state and natural human–computer interaction, first, the computer must be able to analyze and recognize the user’s emotional state, and then the way the computer interacts with the user can be determined and adjusted to have a more intelligent human–computer interaction (Xu and Wan, 2021). At present, there are various researches on emotional information processing of speech signals. It mainly includes the preprocessing of speech signals, the extraction of emotional feature parameters, the establishment and training of emotional models, etc. The classic AI interaction process is shown in Figure 1.

Language is an important means of human communication, and it is the most effective, natural, and direct way for human beings to exchange ideas, thoughts, and emotions. Emotional information can be transmitted not only through speech signals but also through facial expressions and physiological responses. Acoustic representation of language is speech, and speech signals contain a wealth of emotional information. In the case of effective calculations, the impact information contained in the speech is an important source of information for impact calculations. For example, if the same sentence is spoken with different emotions, the emotions expressed are completely different. For example, for the sentence “I will go even if it rains,” under two different emotions of anger and sadness the meanings expressed are very different. This is the so-called “obedience to listen to the sound” truth. Therefore, the voice file of the speaker is collected, analyzed for emotional features contained in speech signals, and this allows the computer to accurately assess the speaker’s emotional state, which has become an important area of research in the field of speech emotion recognition (El-Henawy and Abo-Elazm, 2020; Chatterjee et al., 2021). Speech emotion recognition is to preprocess the speech signal to be recognized, extract feature parameters with emotional tendencies, and through the established emotional model the characteristic parameters are analyzed and identified, a technique used to determine the emotional state of a speaker. By investigating the literature related to speech emotion recognition, the authors analyzed and studied the application of HMM and RBF models in speech emotion recognition, and an improved HMM/RBF hybrid model is proposed starting from the learning process of RBF neural network, introducing dynamic optimal learning rate, improving the stability, and converging the speed of the RBF network.

## Literature Review

In recent years, scholars have researched on speech emotion recognition that originated in the United States. Williams and Stevens were the first to study the emotional information rich in speech, and it was found that the change of the pitch frequency in the speech signal is related to emotion. Deschamps-Berger et al. (2021) recognized it is possible to decode the speech signal frequency contour parameters and recognize speech emotions on four emotions using three different classification algorithms: the nearest neighbor method, the nuclear regression method, and the most probable Bayesian classification method, and obtain the highest recognition rate of about 68%. Sisavath and Yu (2021) analyzed 700 short sentences composed of five emotions (happiness, sadness, anger, fear, and naturalness), and different algorithms were used to differentiate, classify, and recognize the four emotional characteristics parameters (speech speed, shape, base frequency, and energy) and reach an acceptable level of about 70%. Qi et al. (2021) divided speech emotions into positive and negative categories, and two algorithms of linear discrimination and nuclear regression were used compared with Petrushin’s experiment. Li (2021) of the Technical University of Munich developed an open-source toolkit open SMILE, with which the function of batch automatic extraction of speech emotion feature parameters is realized, including common emotional characteristic parameters such as amplitude, fundamental frequency. Zhu et al. (2021) has gradually been Mel cepstral coefficient, and has gradually been widely recognized. Yang et al. (2021) used non-linear dynamic features (NLDs) to classify highly confusing emotions, such as anger and joy. The definitions of basic emotions by different scholars are shown in Table 1 below.

**TABLE 1:** Definitions of basic emotions.

Scholar	Basic emotion
Plutchik	Acceptance, anger, anticipation, disgust, joy, fear, sadness, surprise
Arnold	Anger, aversion, courage, dejection, desire, despair, hate, hope, love, sadness
Ekman	Anger, disgust, fear, joy, sadness, surprise
Frijda	Desire, happiness, interest, surprise, wonder, sorrow
Gray	Rage, terror, anxiety, joy
Izard	Anger, contempt, disgust, distress, fear, guilt, interest, joy, shame, surprise
James	Fear, grief, love, rage

The International Speech Communication Association, abbreviated as ISCA, plays an important role in the study of speech emotions, and scholars from various countries discussed technical problems related to affective computing. ISCA now holds the Eurospeech or Interspeech International Conference every 2 years, and this has greatly contributed to the progress of research in the field of speech emotion recognition. In addition, many universities and scientific research institutions have also done a lot of constructive research work in speech emotion recognition, for example, Cameron University, University of Cambridge, University of Birmingham, and the Japan Research Institute ATR (Ren et al., 2019). In addition, many companies have successively established teams to study emotional computing and artificial intelligence, such as Microsoft, IBM, Google, Sony, etc. The research on speech emotion recognition in China started relatively late, and it is still in its infancy. In recent years, many universities, research institutes, and laboratories have conducted research on speech emotion recognition. The current study of emotion in mature language is based on the study of local culture and language, and Chinese scholars obviously cannot copy foreign research methods, so it is necessary to develop a recognition method that conforms to Chinese speaking habits (Agarwal and Om, 2021). This is because Chinese are not only semantically different from Western countries’ emphasis on light and stress but there are also differences in tone changes. Southeast University was the first to carry out research on speech emotion recognition, among them, Chen (2021) proposed a method of speech emotion recognition using three methods based on principal element analysis, and good recognition results have been achieved. The research team of Zhejiang University established a corpus MASC@CCNT, recorded in Mandarin by 68 young men and women, which included five emotional states, it has laid a solid foundation for the follow-up speech emotion recognition research. Liu and Zhang (2021) proposed a method to measure the effectiveness of emotional feature parameters by using fuzzy entropy. Abbas and Abdullah (2021), taking the prosodic feature as the extracted sentiment feature parameter, performed emotion recognition research on speech signals using two different algorithms, GMM and PNN algorithms, respectively. Professor Bao Canglong of Datong University in Taiwan led an experimental group to establish a Mandarin emotional speech database, the database includes five emotions (Mustaqeem Kwon, 2021). A full-attenuated neural network and a classification and regression tree (CART) algorithm are used to build an emotional model, analyze, and compare the acoustic and prosody features; experiments have shown that acoustic properties have a positive effect on the recognition of speech emotions.

With the development of speech recognition technology, more and more young scholars are also involved in this issue studied and published in the development history (Puri et al., 2022), application direction, technical research content, and key technologies required for emotional computing (Tarnowski et al., 2020). E-learning Lech et al. (2020) is also known as online learning through the Internet, and this also includes reading e-books, distance education is arranged through multimedia playback software, through the Internet, construct virtual online electronic classrooms and online digital libraries and other interactive methods. Liu et al. (2021) conducted research on the semantic aspects of emotional information extracted from images, humans comprehend and perceive images in a subjective way. Shemeis et al. (2021) proposed the Multi-Agent System architecture, the application of this system can realize the recognition of human emotional information, at the same time, it can also express the individual emotions of people in all aspects through the virtual way. Gu Xuejing and Shi Zhiguo proposed an emotional robot based on BDI Agent technology, where they mainly introduced the application of BDI Agent technology in emotional robots by using Agent-based technology to build a speech emotion recognition model, realize that the machine can correctly recognize the input of the external voice. Zhang Yanduo and Wu Hua et al. realized that robot dance can be better combined with music and the robot quickly choreographs its corresponding dance according to the rhythm of the music. This is by extracting the emotional feature parameters in music as the recognition basis, and using the dance system choreographed by robots as the application research object to extract the emotional feature parameters for automatic identification of music, a robot dance choreography system was implemented. Wang S. et al. (2021) proposed an emotion model based on emotion classification. Wang Z. et al. (2021) aimed at how computers can perform emotional calculations A probabilistic model of emotion space is proposed and computer simulation experiments are carried out on it. Wang Y. et al. (2021) adopted the basic theory based on Hidden Markov Model modeling, design an emotion recognition model. Song et al. (2021) summarize and study the current development of the use of speech emotion recognition, the architecture of the recognition model architecture of control robot based on speech emotion is realized, and verified by simulation experiments.

## Research Methods

### Preprocessing of Speech Signals

**FIGURE 2:**
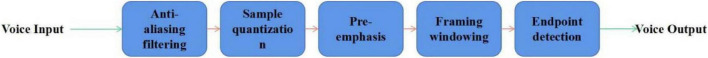
The process of speech signal preprocessing.

The original voice signal is processed to obtain computer-recognizable voice sample data, remove, or reduce the effects of noise in speech signals to obtain ideal speech data for training and recognition of emotion model, this process is the preprocessing of the speech signal. The preprocessing of speech signals is the first step in speech emotion recognition; this generally includes pre-highlighting, windowing for each frame, and endpoint detection, as shown in Figure 2 below.

#### Pre-emphasis

It is difficult to obtain the spectrum of the high-frequency part from the collected original voice signal as this is influenced by the speaker’s nasal radiation and glottic excitation. The high-frequency part generally drops by 6 dB/octave above 800 Hz, therefore, it is necessary to strengthen the high-frequency part of the speech signal, which is of preliminary importance. The high-frequency portion of the pre-stressed speech signal is highlighted and has certain characteristics, and the spectrum is flat, which makes it easy to analyze the spectrum.

In general, first-class FIR high-pass digital filters are used, and the transfer function is as follows:


(1) $$H\,\left(z\right)=1-\alpha \,z^{-1}$$

Here, α is a predictive coefficient, and the value of α ranges from 0.9 to 1.0.

Assuming that the value of the n-sample speech sample is *x*(*n*), then:


(2) y⁢(n)=x⁢(n)-α⁢x⁢(n-1)

Among them, *y*(*n*) is the result after pre-emphasis processing, and is a discrete speech signal.

#### Framing Windowing

The windowing of each speech signal frame is done after the pre-highlighting process. Speech signals have no short-term motion, i.e., in short-term motion the time (10–30 ms) appears to be constant and is determined by the inertial motion of the organ. In addition, although the speech signal is time-varying, it can be regarded as steady-state because the emotional characteristic parameters of the speech signal may appear unchanged in a short period of time. The framing operation of the voice signal is to divide the voice signal into some short segments during processing, an implementation method of weighting using a movable window of finite length.

**FIGURE 3:**
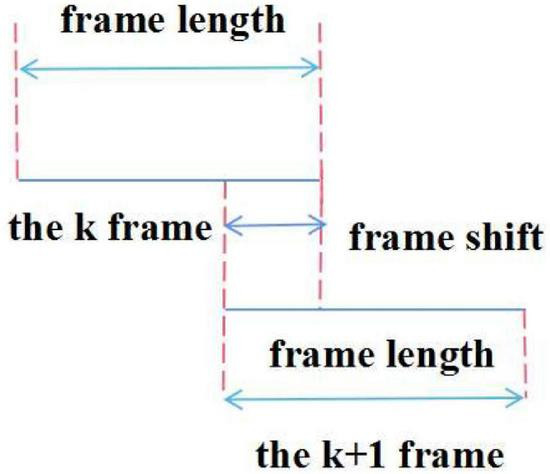
Windowing of speech signals.

However, once this short-term limit is exceeded, the characteristic parameters may change to take some between two adjacent frames’ overlapping part. With overlapping segments, the second half of the previous frame, the first half of the next frame overlapping (also called frame-shift), the continuity of the frame is maintained, which reflects the correlation between the frame and the frame data. The frame-to-frame ratio is generally 0–0.5 and the audible signal range is shown in Figure 3.

For framing processing of speech signals, some kind of operation or transformation is performed on each frame of the speech signal. The expression of the output *x*_*w*_(*n*) after the speech signal *x*(*n*) is framed and windowed is as follows:


(3) $$x_{w}\,\left(n\right)=x\,\left(n\right)*w\,\left(n\right)$$

In the formula, w (n) is the window function.

There are three commonly used window functions: Hamming windows, rectangular windows, and Hanning windows. This article selected the Hamming window function, which can reflect better the spectral characteristics of the speech signal. It is defined as follows:


(4) $$w\,\left(n\right)=\left\{ \begin{array}{c} 0,n\,<0|n>\,N\\ 0.54-0.46\,\cos\left(\frac{2\,\pi \,n}{N-1}\right),0\leq n\leq N \end{array}\right.$$

### Feature Extraction of Speech Signal

Since the original speech signal contains a large amount of emotional information such as intonation, emotion, and rhythm, the extractable emotional feature parameters are also diverse. So if you want to study speech emotion recognition, the first key question is how to get from the many emotional feature parameters, select valid feature parameters, build vectors that reflect personal emotional characteristics, and get better speech emotion recognition rate. An important selection strategy is: to extract the emotional feature parameters that are easier to improve the speech emotion recognition rate as much as possible and reduce those useless redundant information in the speech signal.

#### Pitch Frequency

The authors use the method of short-term autocorrelation function to obtain the fundamental frequency, and the pitch

frequency can be obtained by reciprocating the gene period. The short-term autocorrelation function nR is expressed as follows:


(5) $$R_{n}\,\left(k\right)=\sum_{m=0}^{N_{k}-1}x_{n}\,\left(m\right)-x_{n}\,\left(m-k\right)$$

where *x*(*m*) is the voice signal, *x*_*n*_(*m*) is the nth windowed voice signal intercepted, *N* is the window length, and k is the amount of time delay.

**FIGURE 4:**
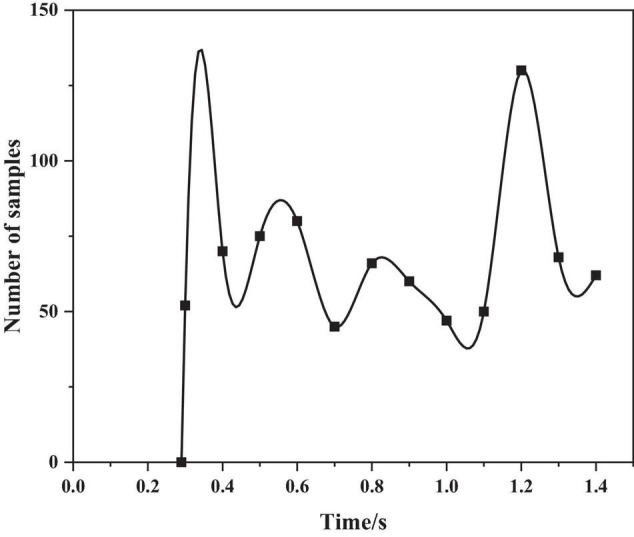
The pitch frequency of anger emotion.

**FIGURE 5:**
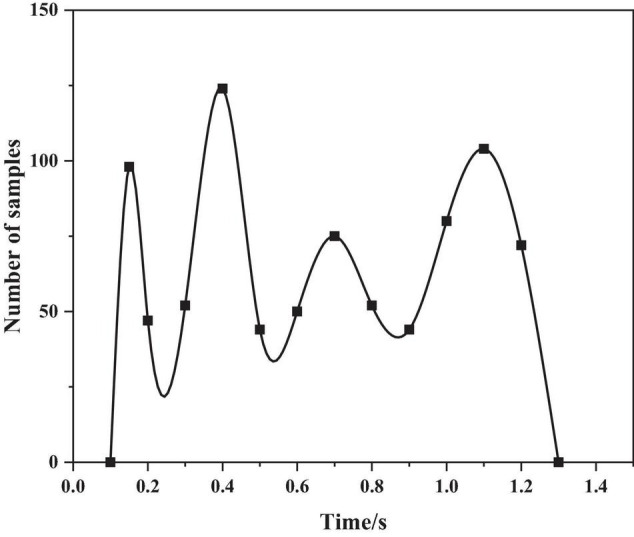
Pitch frequency of happy emotion.

The authors use the sentence “even if it rains” in the CASIA corpus, analyze the pitch frequency under the emotions of anger and happiness, as shown in Figures 4, 5 below.

#### Formant

A formant is a set of resonant frequencies produced by quasi-periodic impulses acting on our vocal tract and it is important to recognize the emotional state and quality of speech in Chinese. Formant is an important parameter of emotional traits in the study of speech emotion recognition. Formant is also an important emotion feature parameter in speech emotion recognition research. The spectral envelope of the speech signal contains formant information, the maximum value of which is the formant; therefore, there is a key point in extracting formant characteristic parameters, i.e., to estimate the spectral envelope of the speech signal. There are two extraction methods for formant characteristic parameters, one is the linear prediction method, and the other is the cepstral method. The authors use the standard complex root method to find the formant parameters. The method is to calculate the root of the prediction error filter A(z) and then the resonance peak can be determined according to the obtained complex root. At the same time, the bandwidth iB and the center frequency iF of the formant can be obtained by decomposing the A(z) polynomial coefficients. The complex roots of the polynomial A(z) can be found using the Newton-Raphson algorithm, where H(z) is related to A(z) as follows:


(6) $$H\,\left(z\right)=\frac{G}{A\,\left(z\right)}-\frac{G}{1-\sum_{i=1}^{p}a_{i}\,z^{-i}}$$

_α_i_ is the linear prediction coefficient, ip = 1,2,…., p is the order, then the relationship is as follows:


(7) $$2\,\pi \,\theta \,F_{i}=e^{-B_{i}\,T\,\pi }-r_{i}$$

where Fi is the formant frequency, the 3 dB bandwidth is Bi, T is the sampling period, and the equations are as follows:


(8) $$F_{i}=\theta_{i}/\left(2\,\pi \,T\right)$$

(9) $$B_{i}=-\ln r_{i}/\pi \,T$$

Since the number of complex conjugate pairs is at most half of the order p in the prediction error filter A(z), and the order p is preset, so the problem of determining the formant to which the pole belongs is not complicated. The speech signal uses the emotionally angry sentence in CASIA “even if it rains,” the vertical point on the power spectrum curve is the position of the formant frequency, and a total of 4 formants were detected. Their formant frequencies Fi and bandwidths Bi are shown in Table 2 below.

**TABLE 2:** Formant frequency and bandwidth values.

	*i* = 1	*i* = 2	*i* = 3	*i* = 4
Formant frequency Fi	610.97	2555.72	4645.03	5958.66
Bandwidth Bi	595.35	698.61	583.90	594.35

**FIGURE 6:**
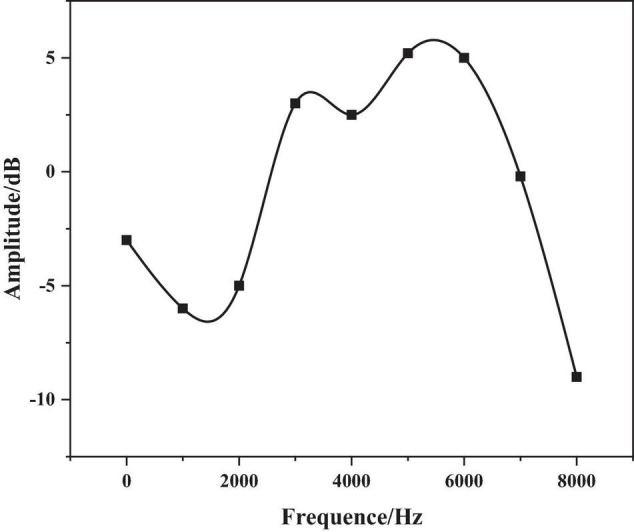
Power spectrum curve of channel transfer function.

The formant frequency is obtained using the LPC root-finding method, as shown in Figure 6 below.

#### Linear Prediction Cepstral Coefficient

In the field of speech emotion recognition, the linear hypothesis coefficient (LPCC) is used as a characteristic of speech emotion recognition emotion, and LPCC can be derived from the linear prediction coefficient (LPC). The main advantage of LPCC is that it can simulate the human vocal tract model well and the excitation information in the process of speech generation can be completely eliminated. At the same time, the calculation of LPCC coefficients is simple and easy to implement. More than a dozen LPCC coefficients can well describe the formant characteristics of speech signals, therefore, a good recognition effect can be obtained in speech emotion recognition. By taking the Fourier transform of the speech signal, the logarithm of the module is taken and then the inverse Fourier transform and the cepstrum of the speech signal are determined.


(10) $$\hat{H}\,\left(z\right)=\ln H\,\left(z\right)\,\sum_{n=1}^{\infty }h\,\left(n\right)\,z^{-n}$$

On substituting Equation (6) into (10), and at the same time taking the derivative of 1z on both sides of the equation, we can get:


(11) $$\left(1-\sum_{i=1}^{p}\alpha_{i}\,z^{-i}\right)=\sum_{n=1}^{\infty }n\,h\,\left(n\right)\,z^{-n+1}-\sum_{i=1}^{p}i\,\alpha \,z^{-i}$$

If the coefficients of z^–1^ in the above formula are equal, respectively, the cepstral coefficient can be obtained from the LPC coefficient. The recursive formula is as follows:


(12) $$\hat{h}\,\left(m\right)=\left\{ \begin{array}{c} \alpha_{n},m=1\\ \alpha_{n}+\sum_{k=1}^{n-1}k\,\hat{h}\,\left(k\right)\,\alpha_{n-k}/m,1<m\leq p+1\\ \sum_{k=1}^{m-1}k\,\hat{h}\,\left(k\right)\,\alpha_{n-k}/m,m>p+1 \end{array}\right.$$

### Hidden Markov Model/Radial Basis Function Hybrid Model Based on Speech Emotion Recognition

#### Mel Frequency Cepstral Coefficient

**FIGURE 7:**
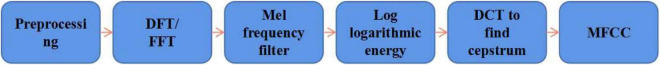
Mel frequency cepstral coefficient extraction process.

Mel frequency Cepstrum coefficient (MFCC) extraction is based on the mechanism of human hearing, that is to say, the frame spectrum of speech is analyzed according to the characteristics of human hearing, and in the presence of spectral distortion and channel noise have a higher recognition accuracy. The process of MFCC coefficient extraction and calculation is shown in Figure 7.

After the original speech signal is preprocessed, each frame time domain signal is obtained.


(13) $$x\left(k\right)=\sum_{n=0}^{N-1}x\left(n\right)e^{-\frac{j\,2\,n\,k}{N}}\left(0<n,k<N-1\right)$$

where *x*(*k*) is the linear spectrum and N is the DFT window width.

To find the logarithmic energy of each filter output, square the spectrum, the energy spectrum, is obtained and then multiplied by a triangular bandpass filter bank. For better robustness, especially to noise and spectral estimation errors, the output of each filter can be taken logarithmically to obtain the logarithmic power spectrum of the corresponding frequency band. Then the inverse discrete cosine transform is performed, thus, L MFCC coefficients are obtained. In general, L takes 12 to 16, and the MFCC coefficients are:


(14) $$C_{n}=\log x\,\left(k\right)\,\cos\left(k-0.5\right)\,n/M,n=1,2\,\ldots \,L$$

#### Hidden Markov Model/Radial Basis Function Hybrid Model

**FIGURE 8:**
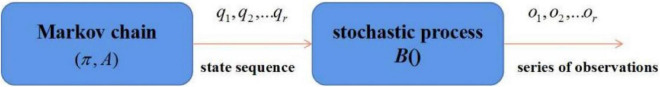
Schematic diagram of the composition of the HMM model.

The composition diagram of the Hidden Markov Model (HMM), called by the same name in Chinese, is shown in Figure 8.

For an effective ensemble RBF neural network for the HMM model, applied to speech emotion recognition, a targeted algorithm must be selected, for which the authors use the Baum-Welch algorithm:

The expression is as follows:


(15) $$\xi_{t}\left(i,j\right)=P\left(q_{t}=i,q_{t+1}=j,O|\lambda \right)$$

It can be deduced from this:


(16) $$\xi_{t}\,\left(i,j\right)=\left[\alpha_{t}\,\left(i\right)\,\alpha_{i\,j}\,b_{j}\,\left(o_{t+1}\right)\,\beta_{t}\,\left(j\right)\right]/P\,\left(O|\lambda \right)$$

The probability of state S_*i*_ in the Markov chain is:


(17) $$\gamma_{t}\left(i\right)=P\left(q_{t}=i,O|\lambda \right)=\sum_{j=1}^{N}\xi_{t}\left(i,j\right)=\alpha_{t}\left(i\right)\beta_{t}\left(i\right)/P\left(O|\lambda \right)$$

This iterative process is repeated to constantly get more optimized parameters, until the parameters are almost unchanged. The model at this time is the final model that meets the actual requirements.

## Analysis of Results

### Experiment Preparation

**FIGURE 9:**
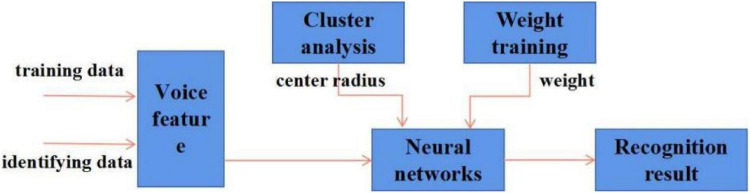
Mixed model neural network training process.

In this experiment, under the Windows operating system environment, the programming simulation is carried out with MATLAB as the working platform. The experimental data uses the CASIA Chinese corpus introduced in Chapter 2, choosing from 5 emotions (happy, sad, angry, surprised, and neutral) with a total of 200 sentences. The experiment adopts the method of ten-fold cross-validation, and the speech sample data is divided into 10 parts, 9 for training and 1 for testing, rotating verification. Then the average of the 10 recognition results is taken as the recognition rate. Sometimes ten-fold cross-validation is performed multiple times; the training process is shown in Figure 9.

In simulation experiments, the sampling frequency of the voice signal is set to 8 KHz and the quantization precision is 16 bit. Using a Hamming window, the frame length is 256 and the frame shift is 128. The simulation results of the preprocessing and feature parameter extraction of the speech signal have been shown in detail in the previous section. The authors select the first and second derivatives of the fundamental frequency 0F and 0F, the first three formants F1, F2, F3, the 10th order LPCC coefficient, and the 10th order MFCC coefficient, a total of 29 parameters for speech emotion recognition.

Due to the different physical meanings and value ranges of the extracted feature parameters, the quantitative relationship between the original data is quite different, it is unreasonable to use these feature parameters directly for identification, and the solution is to normalize these parameters. The code is shown in Table 3 below.

**TABLE 3:** HMM-RBF hybrid model algorithm.

len = length(x); % Calculate vector length
max_x = max(x); % Calculate the maximum value of the vector
min_x = min(x); % Calculate vector minimum
for *i* = 1:1:len
y(i) = (x(i)-min_x)/(max_x-min_x); % normalization processing, [0, 1] numerical interval
End

### Comparison of Recognition Rates

**FIGURE 10:**
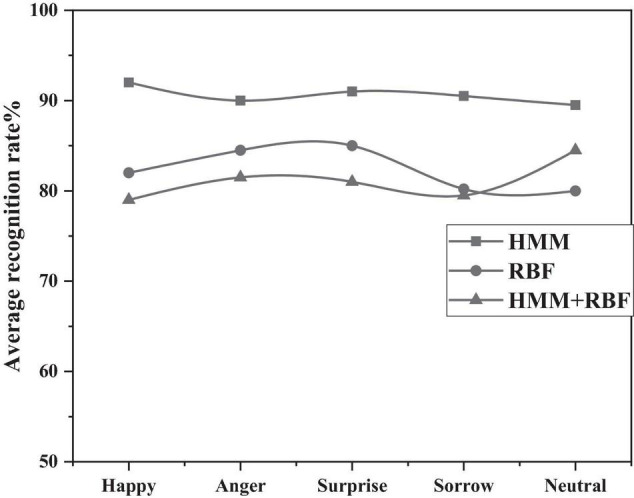
Comparison of recognition rates of three algorithms.

In this context, the HMM/RBF model is compared to the HMM model and the RBF model. As shown in Figure 10 below, from 10 to 81.10% of the HMM model, the average RBF model is 82.84% and the HMM/RBF model is approved 90.98 percent higher. Therefore, the experiments show that the HMM/RBF hybrid model proposed by the authors have better recognition effect.

**FIGURE 11:**
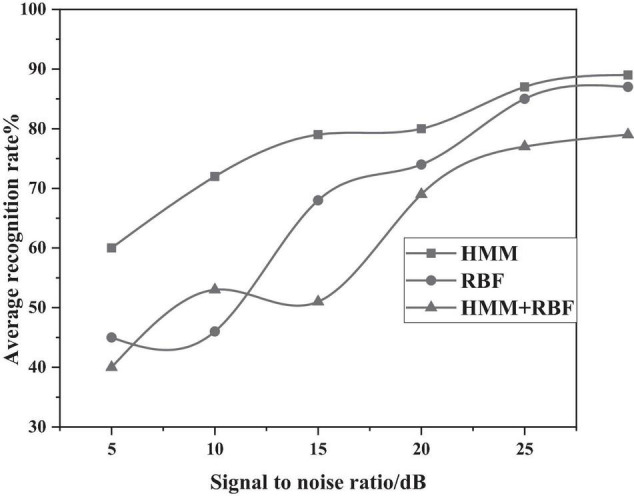
Average recognition rate of three algorithms under different signal-to-noise ratios.

In addition, the authors use the CASIA Chinese sentiment corpus, where the corpus was recorded in a quiet experimental environment to obtain a speech signal without the influence of noise; however, in the process of actually collecting the voice signal, there will inevitably be interference such as background noise. Therefore, white noise with different signal-to-noise ratios were added to conduct the experiment again and verify the anti-noise performance of the established emotion model. The average recognition rate of three emotion models are shown in Figure 11 below, under different SNR environments. It can be clearly seen that, in the case of the HMM/RBF hybrid model with a signal-to-noise ratio of 30 db, the recognition rate can still reach about 89%, a better anti-noise performance than separate HMM and RBF models.

### Comparison of Convergence Speed

Aiming at the learning rate problem of the RBF network in the learning stage, the traditional fixed learning rate is abandoned, the speed of the learning is high, and the speed of the learning is re-calculated every time, and it improves the speed of the RBF network and reduces the time to work. From Table 4, it can be seen that the acceptance rate of the three events has not changed, this is because the initial parameters of the RBF model are the same, and the RBF network after reaching the learning goal is the same, then the recognition rate of the HMM/RBF hybrid model will not change. However, it can be seen that the HMM/RBF hybrid model with the optimal learning rate is better than the HMM/RBF hybrid model with a fixed learning rate, the running time and the number of iterations have been significantly improved, which not only ensures the stability of the network but also improves the operational efficiency.

**TABLE 4:** Convergence speed comparison.

	Learning target	Learning rate	Operation hours	Number of iterations	Recognition rate
HMM/RBF model with fixed learning rate (learning rate 0.01)	0.01	0.01	78.56s	1985	90.98%
HMM/RBF model with fixed learning rate (learning rate 0.02)	0.01	0.02	66.45s	1123	90.98%
HMM/RBF model with optimal learning rate	0.01	Dynamic optimization	40.25s	457	90.98%

A dynamic optimal learning rate is introduced to the RBF network, although this method makes the RBF network in each iteration step recalculate the value of the learning rate and consumes a certain amount of computing time; however, the RBF network is simple and convenient and does not increase the running time of the HMM/RBF hybrid model. In addition, the number of iterations and running time of the improved RBF network are significantly reduced, therefore, the HMM/RBF hybrid model with dynamic optimal learning rate can greatly improve the operation efficiency.

## Conclusion

The authors first introduce the basic theory and knowledge of HMM model and RBF model, the definition of HMM model, and the three basic algorithms are further elaborated. Then they outline the basic knowledge points of the two models, the RBF definition and the learning process. In addition, the concept of “dynamic optimal learning rate” is introduced. The HMM/RBF hybrid model with dynamic optimal learning rate makes the network more stable. Then, the theoretical basis and combination method of the HMM/ANN hybrid model are introduced, and using the strong dynamic time series modeling capabilities of HMM, respectively, and the strong classification decision-making ability of RBF, the two algorithms are combined to improve the recognition rate. Finally, the implementation of the HMM/RBF hybrid model is analyzed in detail, including the training and recognition process. Experiments show that the HMM/RBF hybrid model has better recognition effect than the separate HMM and RBF models and the HMM/RBF hybrid model with the optimal learning rate has a significantly faster convergence rate.

After extensive literature research, it was found that the authors still have many shortcomings that need to be further improved, there are two main aspects:

(1) There are many kinds of speech emotion feature parameters, the authors only select five kinds of feature parameters, including short-term energy, fundamental frequency, formant, LPCC, and MFCC. Further study of other emotional feature parameters is needed, such as selection of the parameters that can best express emotional characteristics, this is one of the issues that need improvement.

(2) The authors use the marked sentiment CASIA Chinese corpus where 20 sentences of text are read aloud by 4 professional actors simulating different emotions; however, this is different from the natural emotional expression of people in daily life. How to collect natural emotional speech signals and build a natural emotion corpus is a problem that needs to be solved.

## Data Availability Statement

The original contributions presented in the study are included in the article/supplementary material, further inquiries can be directed to the corresponding author.

## Author Contributions

Both authors listed have made a substantial, direct, and intellectual contribution to the work, and approved it for publication.

## Conflict of Interest

The authors declare that the research was conducted in the absence of any commercial or financial relationships that could be construed as a potential conflict of interest.

## Publisher’s Note

All claims expressed in this article are solely those of the authors and do not necessarily represent those of their affiliated organizations, or those of the publisher, the editors and the reviewers. Any product that may be evaluated in this article, or claim that may be made by its manufacturer, is not guaranteed or endorsed by the publisher.
